# Effect of epigallocatechin gallate on dental biofilm of *Streptococcus mutans*: An in vitro study

**DOI:** 10.1186/s12903-021-01798-4

**Published:** 2021-09-15

**Authors:** Mor Schneider-Rayman, Doron Steinberg, Ronit Vogt Sionov, Michael Friedman, Miriam Shalish

**Affiliations:** 1 Biofilm Research Laboratory, Institute of Dental Sciences, Faculty of Dentistry, Hebrew University of Jerusalem, Jerusalem, Israel; 2 Department of Pharmaceutics, The Institute for Drug Research, Faculty of Medicine, Hebrew University of Jerusalem, Jerusalem, Israel; 3 Department of Orthodontics, Hadassah Medical Center, Faculty of Dental Medicine, Hebrew University of Jerusalem, Jerusalem, Israel

**Keywords:** Polyphenols, Green-tea, Oral bacteria, Caries

## Abstract

**Background:**

*Streptococcus mutans *(*S. mutans*) plays a major role in the formation of dental caries. The aim of this study was to examine the effect of the green tea polyphenol, epigallocatechin gallate (EGCG), on biofilm formation of *S. mutans*.

**Methods:**

Following exposure to increasing concentrations of EGCG, the planktonic growth was measured by optical density and the biofilm biomass was quantified by crystal violet staining. Exopolysaccharides (EPS) production was visualized by confocal scanning laser microscopy, and the bacterial DNA content was determined by quantitative polymerase chain reaction (qPCR). Gene expression of selected genes was analyzed by real time (RT)-qPCR and membrane potential was examined by flow cytometry.

**Results:**

We observed that EGCG inhibited in a dose-dependent manner both the planktonic growth and the biofilm formation of *S. mutans*. Significant reduction of *S. mutans* biofilm formation, DNA content, and EPS production was observed at 2.2–4.4 mg/ml EGCG. EGCG reduced the expression of *gtfB, gtfC* and *ftf* genes involved in EPS production, and the *nox* and *sodA* genes involved in the protection against oxidative stress. Moreover, EGCG caused an immediate change in membrane potential.

**Conclusions:**

EGCG, a natural polyphenol, has a significant inhibitory effect on *S. mutans* dental biofilm formation and EPS production, and thus might be a potential drug in preventing dental caries.

**Supplementary Information:**

The online version contains supplementary material available at 10.1186/s12903-021-01798-4.

## Background

*Streptococcus mutans (S. mutans)* is the most common pathogen associated with tooth caries [1]. The cariogenic potential of *S. mutans* is associated with its ability to form biofilms on both soft and hard oral surfaces such as the palate, tongue, restorations and teeth [2, 3]. Moreover, it can form biofilms on diverse dental devices including orthodontic brackets and retainers [4]. *S. mutans* produces organic acids upon metabolism of sucrose and other sugars (acidogenicity), and thrives at low pH (aciduricity) [1]. These bacteria rapidly metabolize sugars, such as sucrose and fructose, into glucans or fructans by the extracellular enzymes glucosyltransferases (GTFs), and fructosyltransferases (FTFs). These substances have a crucial role in the virulence of *S. mutans* [5, 6]. The polysaccharides either diffuse into the surrounding environment as extracellular polysaccharides (EPS), or remain associated with the bacteria as pericellular polysaccharides [7, 8]. This EPS-rich matrix is crucial for bacterial adhesion to surfaces and provides mechanical stability for acidogenic and aciduric bacteria, which is essential for dental caries pathogenesis [9–11]. The pathogens’ acid-tolerance properties allow them to colonize the dental surface with plaque formation, inflicting damage to the hard tooth structures, making the teeth vulnerable to decay and to caries formation.

*S. mutans* possesses three different GTFs, encoded by *gtfB*, *gtfC*, and *gtfD*, each synthesizing unique proportion of water-soluble and -insoluble glucan polymers. The glucans, along with glucan-binding proteins such as *GbpA* and *GbpB*, promote a persistent adhesion and accumulation on tooth surfaces. The second type of extracellular enzyme responsible for metabolizing sugars is FTF, encoded by the *ftf* gene, which is responsible for fructan synthesis [12, 13].

A biofilm is a community of microbial cells, which has been attached to a surface, enwrapped in a matrix of polysaccharide material [14]. The oral biofilms consist of various non-cariogenic and cariogenic bacteria embedded in an extracellular matrix composed of bacterial enzymes (e.g., GTFs, FTF), polysaccharides, nucleic acids, proteins, bacteria-secreted compounds, salivary components including salivary enzymes and food remnants, as well as other host constituents [15].

Currently, common preventive means to inhibit oral diseases include aggressive chemical agents, such as chlorhexidine and antibiotics, which have various undesired side effects, including tooth staining, mucosal erosion, taste disturbance and bacterial resistance [16–18]. Therefore, naturally occurring compounds, such as green tea polyphenols, have attracted much attention.

Due to divers health benefits, there has been a significant increase in consumption of green tea among various cultures, making it one of the most popular beverage in the world [19]. Green tea, produced from *Camellia sinensis* (*C. sinensis*) leaves, has a high concentration of polyphenols, in particular catechins, which possess anti-oxidant properties. The leaves of *C. sinensis* undergo minimal oxidation during processing and thus preserve their anti-oxidant and anti-bactericidal properties [17]. The major tea catechins include epigallocatechin 3-gallate (EGCG), epigallocatechin (EGC), epicatechin (EC), epicatechin 3-gallate (ECG), and catechin (C) [20, 21]. These polyphenols were found to have anti-microbial traits and can inhibit a wide range of gram-positive and gram-negative bacteria in vitro [22, 23].

EGCG has been shown to disrupt EPS and biofilm formation of *S. mutans*, by suppressing *gtfB*, *gtfC* and *gtfD* genes [24, 25]. Green tea polyphenols, especially EGCG, have the ability to interfere with quorum sensing (QS), which is essential for biofilm formation by different bacteria [26, 27]. Quorum sensing is considered a potential target of anti-microbial compounds. One of the mechanisms of tea catechins to damage bacteria is binding to the bacterial cell membrane, which prevents the ability of the bacteria to bind to each other and to form biofilm [28]. In vivo studies have shown that green tea mouthwash has the ability to inhibit *S. mutans* biofilm formation on tooth surface when given to dental population [29–31]. These studies raised the importance of green tea polyphenols as natural anti-microbial compounds, which can be safe for use and prevent dental diseases. Since EGCG is a major polyphenol of tea extracts and EGCG tablets are provided as a natural supplement, we wanted to study the effect of this EGCG source on *S. mutans* viability, EPS production and biofilm formation in vitro. It is also worthwhile to assay the membrane potential since it regulates metabolism, bacterial cell division, pH homeostasis, and membrane transport [32]. The aim of the present study was to examine the action mechanisms of EGCG on *S. mutans* with specific emphasize on planktonic growth and biofilm formation. Here we show that EGCG has both growth inhibitory and anti-biofilm activities. The minimum biofilm inhibitory concentration (MBIC) was lower than the minimum growth inhibitory concentration (MIC), suggesting a direct anti-biofilm effect. Some mechanistic insights are presented.

## Materials and methods

### Materials

One EGCG tablet (Source Naturals, Scotts Valley, CA, USA) containing 700 mg EGCG, was dissolved in 10 ml of DDW by a 1 h shaking at 4 °C. The stock EGCG solution (70 mg/ml) was sterile filtered through a filtration device with a pore size of 0.22 μm (Merck Millipore, Darmstadt, Germany), and diluted 1:1 in brain heart infusion (BHI) broth (Acumedia, Neogen, Lansing, MI, USA) concentrated x2 to get a final BHI x1 concentration. Then serial dilution was done in BHI to achieve final concentrations of 0.13–17.6 mg/ml EGCG. Since the effective concentrations were in the range of 0.55–4.4 mg/ml, only data obtained with these concentrations are presented. The working solutions were used fresh. For planktonic growth, the bacteria were incubated in BHI, while for biofilm formation BHI was supplemented with sucrose to a 2% final concentration (BHIS) [33]. The diluted EGCG solutions in BHI/BHIS were filtrated through a filtration device with a pore size of 0.22 μm before use to remove any precipitates. Control bacteria received the same incubation conditions without EGCG (see below).

### Planktonic growth and biofilm formation

*Streptococcus mutans* (*S. mutans*) UA159 from the stock of the Biofilm Research Laboratory, was grown as monospecies culture. Before each experiment, a frozen stock of *S. mutans* was inoculated in brain heart infusion (BHI) broth, at a ratio of 1:100, and incubated at 37 °C overnight in the presence of 5% CO_2_ until reaching an optical density (OD)_600_ of 1.2 [34, 35]. For planktonic growth, the overnight *S. mutans* cultures were diluted 1:10 in BHI in the absence or presence of various concentrations of EGCG (0.55–4.4 mg/ml), and the resulting OD at 595 nm was measured after a 24 h incubation using the Infinite M200 PRO plate reader (Tecan Trading AG, Switzerland). The percentage of bacteria in planktonic phase was calculated by dividing the OD of treated samples by OD of control samples, multiplied by 100, after subtracting the background OD of an EGCG solution in BHI in the absence of bacteria. MIC_50_ and MIC_80_ were defined as the concentration resulting in 50 and 80 decrease in bacterial viability, respectively.

For biofilm formation, the overnight *S. mutans* cultures were diluted 1:10 in BHI supplemented with 2% sucrose (BHIS) in the absence or presence of various concentrations of EGCG (0.55–4.4 mg/ml), and the biofilms were allowed to develop for 24 h at 37 °C in 95% air/5% CO_2_. In parallel, BHI without bacteria in the absence or presence of EGCG was used to measure any background signals caused by EGCG in the assays used (see below). The setup for biofilm formation was as follows: 200 µl of BHIS containing different concentrations of EGCG and 20 µl of *S. mutans* were added to each well of a 96 flat-bottomed well tissue culture plate (Corning, NY, USA). Each EGCG concentration was tested in triplicates. MBIC_50_ and MBIC_95_ were defined as the concentration resulting in 50 and 95% decrease of biofilm formation, respectively.

### Quantification of biofilm biomass by crystal violet (CV) staining

The biofilm biomasses were quantified using the crystal violet assay as described [36], with slight modifications. The biofilms formed in the 96-well tissue culture plates after treatment with EGCG were carefully washed twice with PBS to remove unbound bacteria and to obtain a clean biofilm. 200 µl of a 0.1% Crystal Violet solution that was prepared from a 0.4% Gram’s crystal violet solution (Merck, Darmstadt, Germany) by dilution with DDW, were added to each well. After 20 min incubation at room temperature (RT), the stained biofilms were washed twice with DDW and left to dry overnight at RT. At the following day, 200 µl of 33% acetic acid were added to dissolve the stain for 15 min at RT. After dissolving the stain, 150 µl were transferred to a new 96-well tissue culture plate and quantified spectrophotometrically by measuring the absorbance at 595 nm using the M200 plate reader. The percentage biofilm formation was calculated by dividing the OD of treated samples by OD of control samples, multiplied by 100.

### Confocal laser scanning microscopy (CLSM)

The CLSM was performed to determine the biofilm depth, the presence of extracellular polysaccharides (EPS), and the amounts of live/dead bacteria. To label the EPS in the biofilms, 1 µl of a 1 mM Alexa Fluor 647-labeled Concanavalin A (ConA) conjugate solution (Molecular Probes, Life Technologies, Carlsbad, California, USA) was added to the samples during the incubation period with or without EGCG. After incubation, the biofilms were washed twice with 200 µl PBS and stained with 50 µl of live/dead (SYTO 9/propidium iodide (PI)) BacLight fluorescent dye (Molecular Probes, Life Technologies, Carlsbad, California, USA) for 20 min in the dark at RT. Live bacteria showed green fluorescence, while dead bacteria emitted red fluorescence. The stained biofilms were inspected under a Nikon Spinning Disk microscope (Nikon Corporation, Tokyo, Japan) connected to Yokogawa W1 Spinning Disk (Yokogawa Electric Corporation, Tokyo, Japan) [37]. Optical sections were acquired at spacing steps of 5 μm intervals from the surface through the depth of the biofilm. A three-dimensional image of the microbes and EPS distribution within the biofilms was constructed using the Nikon Imaging Software (NIS- Elements). The NIS elements software was used to quantify the fluorescence intensity in each biofilm layer.

### Quantitative real-time (qRT)-polymerase chain reaction (PCR) analysis

The assay was performed similarly as described [38, 39]. Biofilms were allowed to form in 6-well tissue culture plates (Corning). Each sample consisted of 400 µl of an overnight culture of *S. mutans* (OD = 1.2), mixed with 4 ml BHIS containing different concentrations of EGCG. After a 24 h incubation, 1 ml of Tri-Reagent (Sigma-Aldrich, St. Louis, MO, USA) was added to the washed biofilms to extract the total RNA from the biofilms. The biofilms were scraped into the Tri-Reagent solution with the help of a sterile cell scraper, and the fluid was transferred into 2 ml sterile screw tubes containing 200 µl acid-washed glass beads followed by cell disruption in a Fast Prep Cell Disrupter (Bio 101, Savant Instruments, Inc., NY, USA). After centrifugation to remove the glass beads, the supernatant was transferred to a new Eppendorf tube and 200 µl of chloroform (Bio-Lab, Jerusalem, Israel) were added to each sample, followed by vigorous vortex for 15 s. After 15 min at RT, the samples were centrifuged at 13,000 rpm for 15 min at 4 °C, and the upper phase (400 µl) was transferred to a new tube. 400 µl of isopropanol was added to the upper aqueous phase to precipitate the RNA. The samples were allowed to stand at RT for 30 min before centrifugation at 13,000 rpm for 30 min at 4 °C. After centrifugation, the pellet was washed with 75% ethanol, and after recentrifugation, the pellet was allowed to dry for 30 min at RT. The dried RNA was resuspended in ultrapure water (UPW). The purity and concentration of the RNA were determined using a Nanodrop ND-1000 Instrument (Wilmington, DE, USA). The purified RNA was reverse transcribed into cDNA using the qScript cDNA synthesis kit (Quanta Biosciences, Beverly, MA, USA) and PCR amplification was done in a CFX96 BioRad Connect Real-Time PCR apparatus using Power Sybr Green Master Mix (Applied Biosystems) on 2 ng cDNA in the presence of 300 nM forward/reverse primer sets (Additional file 1: Table 1). PCR conditions included an initial heating at 50 °C for 2 min, an activation step at 95 °C for 10 min, followed by 40 cycles of amplification (95 °C for 15 s, 60 °C for 1 min). Calculations were done according to the 2^−ΔΔCt^ method, where 16 and 23 S rRNA served as internal standards. Gene expression was expressed in relative values, setting the expression level of the control samples to one.

### Membrane potential

The membrane potential of untreated and EGCG-treated planktonic *S. mutans* was measured using the cationic dye 3,3′-diethyloxacarbocyanine iodide (DiOC_2_(3); Molecular Probes, Eugene, OR, USA) by flow cytometry according to the manufacturer’s instructions. DiOC_2_(3) exhibits green fluorescence in all bacterial cells, but the fluorescence shifts toward red emission at higher membrane potential values. Briefly, an overnight culture of *S. mutans* was resuspended in PBS to an OD of 0.3. The bacterial suspension was divided into 1 ml aliquots with different concentrations of EGCG and stained with 10 µl of 3 mM DiOC_2_(3) for 30 min in the dark. The samples were analyzed by flow cytometry (LSR-Fortessa flow cytometer, BD Biosciences), using the 488 nm excitation laser and collecting the data using the green (530nm) and red (610/620 nm) filters [40]. In addition, we measured the forward scatter (FSC) and the side scatter (SSC) of the untreated and EGCG-treated bacteria. FSC detects light scatter along the path of the laser and its intensity is proportional to the diameter of the cell. SSC measures light scatter at a ninety-degree angle relative to the laser and provides information about the granularity of the cell.

### High-resolution scanning electron microscopy (HR-SEM)

Untreated and EGCG-treated planktonic *S. mutans* after 2 h incubation were fixed in 4% glutaraldehyde (MERCK, Darmstadt, Germany) in DDW for 2 h and then washed in DDW and let dry. Thereafter, the samples were coated with iridium and visualized using a Magellan 400 L High Resolution Scanning Electron Microscope (HR-SEM) at 10,000×–50,000× magnifications [33].

### Statistical analysis

Experiments were performed at least three times in triplicates and statistical significance of treated samples versus control samples was analyzed using the Student’s t test in the Microsoft Excel software, with a *p* value of less than 0.05 considered significant.

## Results

### Anti-bacterial and anti-biofilm activity of EGCG

**Fig. 1:**
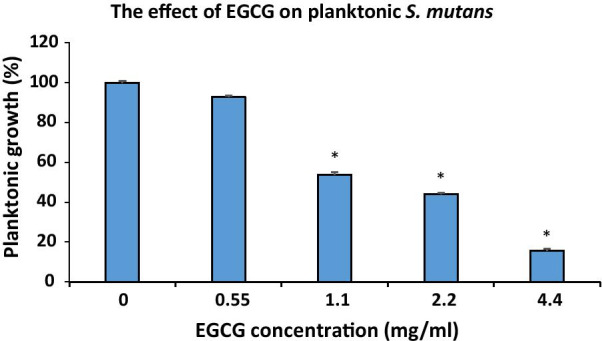
The effect of epigallocatechin gallate (EGCG) on planktonic growth of *S. mutans*. Planktonic growth of *S. mutans* treated with different concentrations of EGCG for 24 h as measured by optical density (OD) at 595nm. n = 3; *p < 0.05 compared with the control samples

We first studied the effect of EGCG on the planktonic growth of *S. mutans* and observed that EGCG reduced the planktonic growth of *S. mutans* in a dose-dependent manner, with a significant growth inhibition at an EGCG concentration of 1.1 mg/ml (50 ± 2%) and 2.2 mg/ml (56 ± 2%) with a maximal effect at 4.4 mg/ml (84 ± 2%) (Fig. 1). Therefore, the MIC_50_ and MIC_80_ of EGCG was 1.1 mg/ml and 4.4 mg/ml, respectively.

**Fig. 2:**
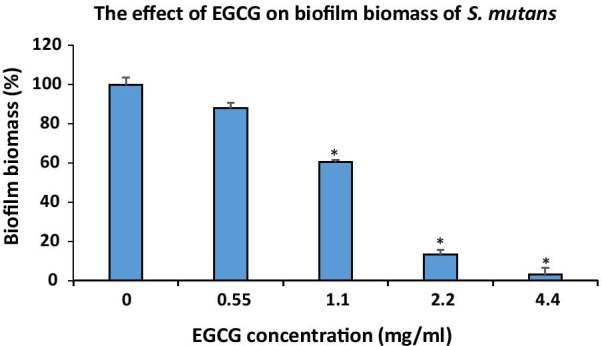
The effect of epigallocatechin gallate (EGCG) on biofilm biomass of *S. mutans*. Biofilm biomass of *S. mutans* after a 24 h incubation with different concentrations of EGCG, as determined by crystal violet (CV) staining measured at an optical density (OD) of 595 nm. n = 3; *p < 0.05 compared with the control samples

We next studied the effect of EGCG on biofilm formation. To this end, *S. mutans* was incubated for 24 h with increasing concentrations of EGCG, and the biofilm biomass was quantified by CV staining. It is evident from Fig. 2 that EGCG significantly decreased the biofilm biomass of *S. mutans* in a dose-dependent manner, starting from 1.1 mg/ml EGCG, with the most significant effect at a concentration equal to 2.2 mg/ml and above, when compared to the control. At 2.2 mg/ml EGCG the biofilm mass was reduced by 86 ± 3%, and at 4.4 mg/ml EGCG there was almost no biofilm (3 ± 1% compared to control bacteria) (Fig. 2). Therefore, the MBIC_50_ and MBIC_95_ of EGCG was 1.1 mg/ml and 4.4 mg/ml, respectively. At 2.2 mg/ml and 4.4 mg/ml EGCG the biofilm inhibitory effect was stronger than the growth inhibition (compare Fig. 2 with Fig. 1), suggesting that EGCG has a specific anti-biofilm activity.

**Fig. 3:**
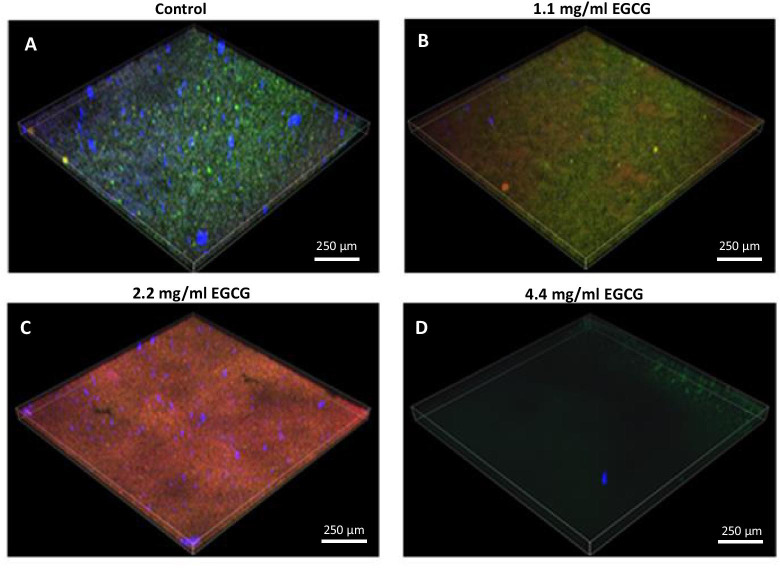
Confocal laser scanning microscopy (CLSM) images of SYTO 9/propidium iodide (PI)-stained biofilms. Epigallocatechin gallate (EGCG) reduced the number of live bacteria and the amount of exopolysaccharides (EPS) in a dose-dependent manner. Computerized 3D reconstruction of the biofilm layers after treating *S. mutans* with different concentrations of EGCG for 24 h, as recorded by CLSM and generated by the Nikon Imaging Software (NIS-elements). **a** Control. **b–d** Increasing concentrations of EGCG (mg/ml). A representative sample of each treatment is shown. Green color represents live cells, red color represents dead cells and blue color represents the EPS

The biofilm mass of untreated and EGCG-treated biofilms was further studied by live/dead SYTO 9/PI staining together with EPS staining, a technique where live bacteria show green fluorescence, dead bacteria emit red fluorescence and EPS stained with Alexa Fluor^647^-conjugated ConA appears as blue fluorescence. The reconstructed CSLM 3D images show that EGCG reduced the number of live bacteria and the amount of EPS in a dose-dependent manner (Fig. 3). An increase in dead bacteria was observed in the samples treated with 1.1 and 2.2 mg/ml EGCG (Fig. 3B, C). At 4.4 mg/ml EGCG there were almost no bacteria seen in the biofilms (Fig. 3D).

**Fig. 4:**
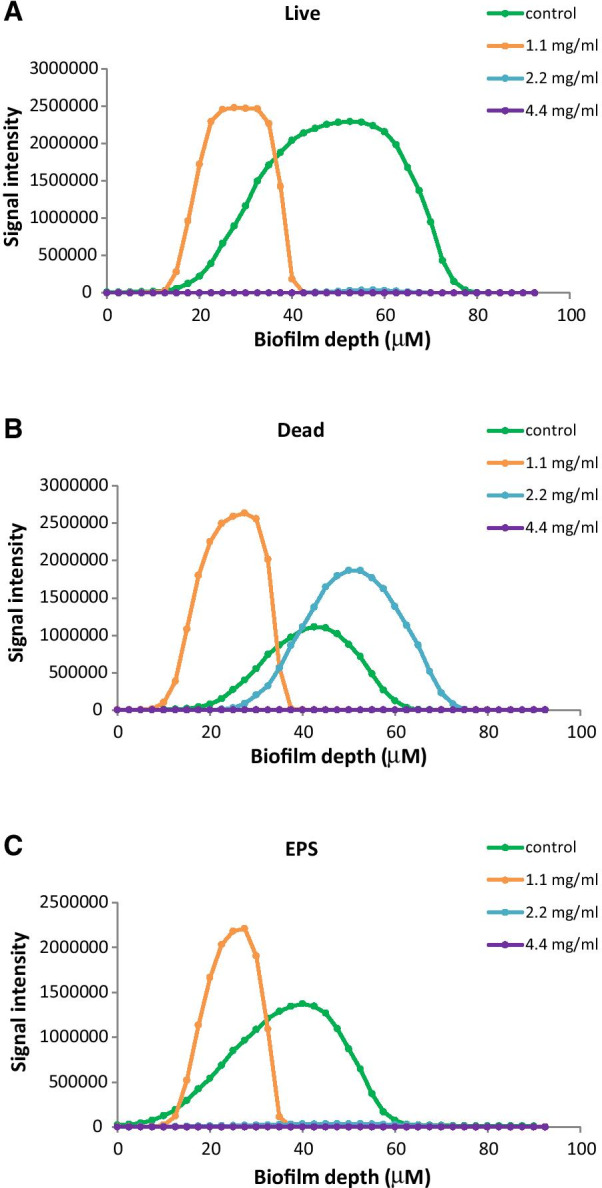
Quantification of the SYTO 9/propidium iodide (PI) staining of biofilms formed in the absence or presence of epigallocatechin gallate (EGCG). Quantification of the fluorescence intensity of each biofilm layer after treating *S. mutans* with increasing concentrations of EGCG for 24 h. **A** Live bacteria stained by SYTO 9. **B** Dead bacteria stained by PI. **C** Exopolysaccharides (EPS) production stained by Alexa Fluor^647^**-**conjugated ConA

The three fluorescence intensities in the different layers of the images in Fig. 3 were quantified using the NIS elements software. According to Fig. 4A, the depth of the live biofilm was reduced from 60 microns in the control samples to 20 microns in the samples treated with 1.1 mg/ml EGCG, with a concomitant 46% reduction in the total green fluorescence intensity. Only remnants of live bacteria were seen in the samples treated with 2.2 and 4.4 mg/ml EGCG (Fig. 4A). We observed a higher PI staining of dead bacteria in the 1.1 and 2.2 mg/ml EGCG treated group in comparison to control (Fig. 4B). At 4.4 mg/ml EGCG there were almost no bacteria left, which explains why there were no significant PI staining. The EPS staining showed similar pattern to the SYTO 9 staining, with 21% reduction in signal intensity at 1.1 mg/ml EGCG in comparison to control. There was no significant EPS signal in the 2.2 and 4.4 mg/ml EGCG treated samples (Fig. 4C), which goes along with the strong reduction in the number of live bacteria (Fig. 4A).

**Fig. 5:**
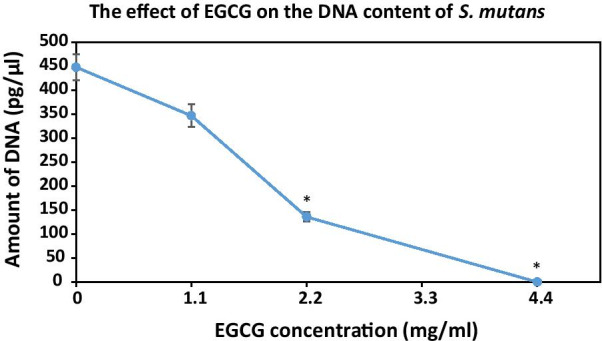
The effect of epigallocatechin gallate (EGCG) on the DNA content of *S. mutans* biofilm. Quantitative polymerase chain reaction (qPCR) analysis of the DNA content in *S. mutans* biofilms after treatment with different concentrations of EGCG for 24 h. n = 3; *p < 0.05 compared with the control samples

The anti-biofilm effect of EGCG was further demonstrated by determining the amount of DNA in the resulting biofilms. The DNA content of *S. mutans* biofilm formed after treatment with EGCG was found to be significantly decreased at concentrations of 2.2 mg/ml and above, when compared to the control (Fig. 5).

### EGCG reduced the expression of biofilm-related genes

**Fig. 6:**
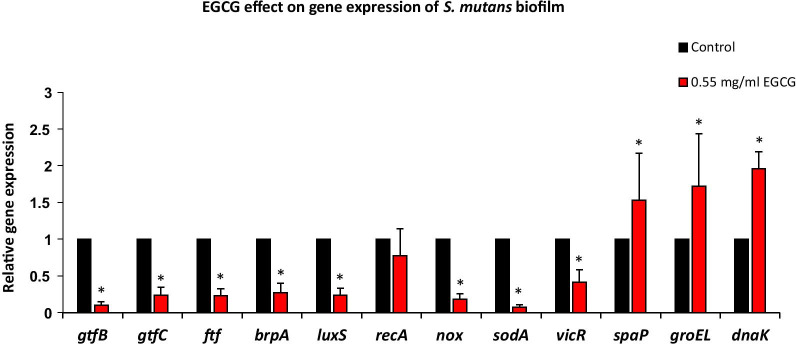
The effect of epigallocatechin gallate (EGCG) on gene expression in *S. mutans* biofilm. Real-time PCR analysis of various genes involved in biofilm formation and oxidative stress after a 24 h treatment of *S. mutans* with EGCG (0.55 mg/ml) n = 3; *p < 0.05 compared with the control samples. The relative expression levels of the genes analysed by real-time PCR was normalized against *16S* rRNA and *23S* rRNA that served as internal standards

Since we observed that EGCG reduced the biofilm formation of *S. mutans*, we questioned whether this compound affects gene expression of biofilm-related genes. Biofilms of *S. mutans* were treated with a sub-inhibitory concentration of EGCG (0.55 mg/ml) for 24 h and the RNA was extracted from the resulting biofilms. Our data (Fig. 6) showed several changes in the gene expression pattern. There was a strong down-regulation of the *gtfC, gtfB* and *ftf* genes involved in EPS production (77–90% reduction), and in the *brpA* gene that regulates biofilm formation (73 ± 13% reduction). In addition, EGCG caused a strong down-regulation of the quorum sensing genes *vicR* and *luxS* (59–76% reduction) and of the genes *nox* and *sodA* involved in the protection against oxidative stress (82–92% reduction). On the other hand, there was a significant upregulation of the virulence gene *spaP* and of the stress response heat-shock protein genes *groEL* and *dnaK* (1.5–2 fold increase).

### EGCG caused an immediate hyperpolarization of the bacterial membrane

**Fig. 7:**
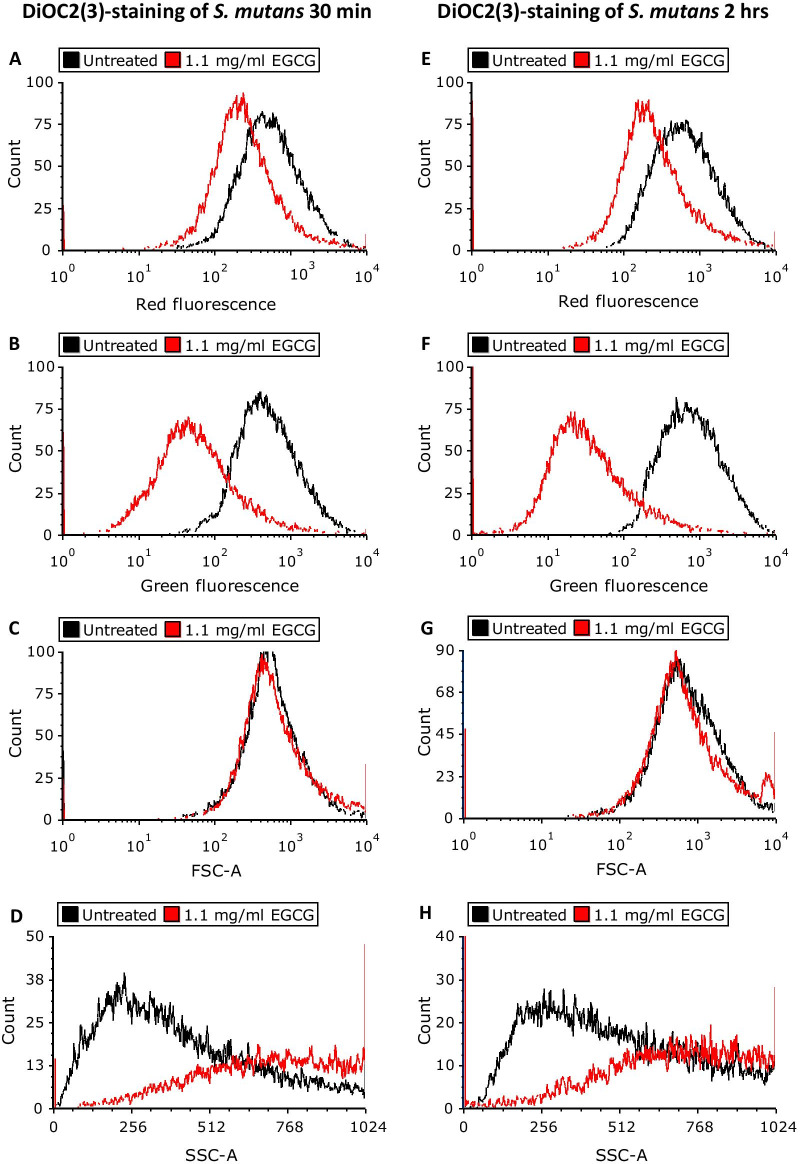
The effect of epigallocatechin gallate (EGCG) on membrane potential of planktonic *S. mutans.* DiOC_2_(3) staining of *S. mutans* treated with 1.1 mg/ml EGCG, as determined by flow cytometry. **A.** Red fluorescence (30 min). **B** Red fluorescence (2 h). **C** Green fluorescence (30 min). **D** Green fluorescence (2 h). **E** Forward scatter area (FSC-A) (30 min). **F** FSC-A (2 h). **G** Side scatter area (SSC-A) (30 min). **H** SSC-A (2 h). Similar results were obtained with 2.2 mg/ml and 4.4 mg/ml EGCG

Since the membrane potential affects many of the bacterial functions [32], it was prompting to analyze the effect of EGCG on this parameter. The membrane potential of planktonic *S. mutans* was measured 30 min and 2 h after exposure to EGCG using the DiOC_2_(3) reagent on flow cytometry. The green fluorescence is an indication for the amount of dye taken up by the bacteria, while the red fluorescence is increased upon higher membrane potential. According to Fig. 7, EGCG-treated bacteria showed a slight reduction in the red fluorescence in comparison to untreated bacteria, that was not proportional to the strong reduction in the green fluorescence. This means that less dye is incorporated in the EGCG-treated bacteria than the untreated bacteria, yet the red/green fluorescence ratio is increased. The latter observation suggests that EGCG induces hyperpolarization of the membrane potential. There was no significant difference between the two different incubation times, suggesting that the immediate hyperpolarization of the membrane by EGCG was maintained over time. Notably, EGCG caused a significant shift in the side scatter (SSC) on flow cytometry (Fig. 7G, H) without any significant effect on the forward scatter (FSC). The increase in SSC suggests that EGCG leads to an irregular structure of the bacteria.

**Fig. 8:**
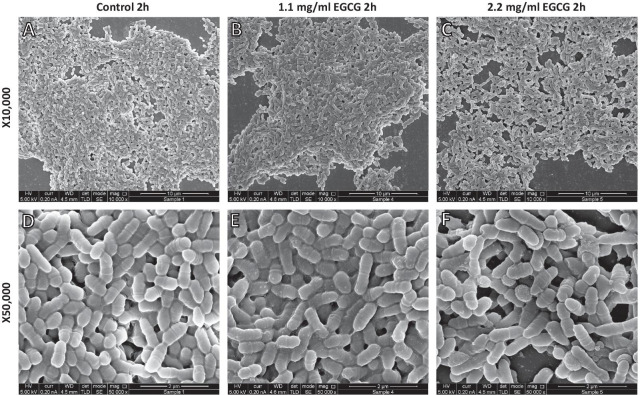
High resolution scanning electron microscope (HR-SEM) images of epigallocatechin gallate (EGCG)-treated planktonic *S. mutans*. Planktonic *S. mutans* was exposed to different concentrations of EGCG for 2 h, fixed and processed for HR-SEM imaging. **A**, **D** Control. **B**, **E** 1.1 mg/ml EGCG. **C**, **F** 2.2 mg/ml EGCG. **A**–**C** x10,000 magnification. **D–F** x50,000 magnification

We, therefore, performed HR-SEM imaging on planktonic *S. mutans* incubated in the absence (control) or presence of EGCG for 2 h. The EGCG-treated bacteria appeared with nano-scale dotted structures on their surfaces, in contrast to the smooth surface of control bacteria (Fig. 8). It could be that these are EGCG-induced protein precipitated aggregates.

## Discussion

Oral biofilm is associated with a variety of oral diseases, inflicting the dental population by inducing caries, gingivitis, and periodontal diseases. Conventional treatment and prevention of oral diseases include chemical agents such as chlorhexidine and antibiotics, which have various undesired side effects such as teeth discoloration and bacterial resistance [16–18]. Recently, natural compounds have been proposed as novel treatment options for oral diseases, in an effort to avoid side effects derived from common drug delivery means [29, 41]. Green tea, which is one of the most popular beverages in the world, contains high concentrations of anti-oxidants, one of them is EGCG.

Taylor et al. [23] studied the biological activity of green tea components including EGCG. They showed that the green tea components have anti-microbial and anti-cariogenic effects, with therapeutic potential for preventing periodontal diseases. Xu et al. [24] observed similar attributes of EGCG by demonstrating its anti-bacterial and anti-cariogenic properties against *S. mutans*. Hirasawa et al. [42] also demonstrated a bacteriocidal effect of EGCG using different techniques.

Our study demonstrates that EGCG has the ability to inhibit the planktonic growth of *S. mutans*. We also demonstrate an inhibitory effect of EGCG on *S. mutans* biofilm formation in a dose-dependent manner, with a MIC_80_ at 4.4 mg/ml of EGCG. The EGCG-induced reduction in biofilm formation was demonstrated by crystal violet staining, quantification of DNA content and CLSM. CLSM imaging also showed that EGCG decreased the biofilm thickness, reduced the number of viable bacteria, increased the number of dead bacteria, and inhibited EPS production. Although at 2.2 mg/ml and 4.4 mg/ml the survival of planktonic bacteria was 44 ± 2% and 16 ± 2%, respectively, the ability of the bacteria to form a biofilm was only 14 ± 3% for 2.2 mg/ml and 3 ± 1% for 4.4 mg/ml EGCG in comparison to control bacteria, suggesting a specific anti-biofilm effect of EGCG in addition to its anti-bacterial activity.

Our assumption was that the reduced production of EPS by EGCG could be due to a down-regulation of genes responsible for EPS production, such as *gtfB, gtfC* and *ftf*. Banas [7] showed that *S. mutans* strains inactivated in one or more *gtf* genes, had diminished virulence when tested in rodent models of caries. Xu et al. [25] demonstrated that EGCG can suppress *gtfB and gtfC* genes. Our results support these findings by demonstrating that the sub-toxic concentration of 0.55 mg/ml EGCG significantly inhibited *gtfC, gtfB* and *ftf* gene expression by 77–90% compared to control. It should be noted that EGCG also caused a strong down-regulation of *nox* and *sodA* genes (82–92% reduction) which are involved in the protection against oxidative stress, and increased the expression of the stress response genes *groEL* and *dnaK* (1.5–2 fold increase).

We assumed that one of the possible mechanisms of biofilm inhibition by EGCG is an effect on the membrane potential of *S. mutans*. When studying this parameter using the DiOC_2_(3)-membrane potential sensitive dye on flow cytometry, our results show higher red/green fluorescence ratio, indicative for a hyperpolarized membrane potential. Remarkably, we observed that EGCG treatment of *S. mutans* showed a right shift in the side scatter (SSC) suggesting an irregular structure of the bacteria. There was no significant difference between a 30 min and a 2 h incubation time, suggesting an immediate effect of EGCG on the bacterial structure. HR-SEM images show several dotted structures on the membranes of EGCG-treated bacteria. It is known that EGCG interacts with proteins [43], inactivates enzymes [44] and can also precipitate out proteins [45]. Thus, the dotted structures observed on the EGCG-treated bacteria seem to be protein aggregates that can alter various bacterial functions. It is likely that the protein-modulating action of EGCG affects the activities of ion transporters resulting in the membrane hyperpolarization observed after exposure to EGCG. The appearance of the dotted structures on the bacterial surface may contribute to the altered SSC observed on flow cytometry, since they will cause a different reflection of the light beam.

Treating bacterial diseases by natural compounds that may reduce drug-resistance and lower adverse side effects is a common goal for clinicians and researchers alike. Our in vitro study was performed as a step forward to develop a strategy to prevent biofilm formation on both biological materials and orthodontic devices. While our findings support the use of green tea derived EGCG in the fight against oral bacteria and carries prevention in vitro, further clinical trials are needed to demonstrate this effect in vivo. This study was performed in an in vitro model of *S. mutans* biofilm formation, while the next step will be testing the EGCG in a multi-biofilm model, as dental caries is a complex multispecies process involving different bacteria species and host components. The anti-biofilm activity of EGCG is nevertheless important, and further clinical studies are required to examine how this trait of EGCG can be used to prevent oral bacterial biofilm formation in the oral cavity. Several studies have indeed suggested that EGCG can be a good candidate for this purpose [46–49]. In this study, we have further shown some mechanistic insights showing that the EGCG has an anti-biofilm activity (e.g., direct inhibition of expression of genes involved in biofilm formation) that differs from the anti-bacterial activity (e.g., alterations in membrane potential and EGCG-induced precipitation of proteins that leads to the inactivation of their biological activities).

## Conclusions

We have shown that EGCG has both anti-bacterial and anti-biofilm activities against the cariogenic bacteria *S. mutans*. These two activities are distinct being mediated by different action mechanisms. Although reduced biofilm formation can be caused by reduced bacterial growth, EGCG affects the expression of genes regulating biofilm formation. Our data support the consensus that EGCG is a potential natural compound for the prevention of oral diseases such as tooth decay. 

## Supplementary Information

**Additional file 1**. Table 1: The primers used for real-time PCR.

## Data Availability

The raw datasets used during the present study are available from the corresponding author upon reasonable request.

## Abbreviations

CLSM: Confocal laser scanning microscopy
CV: Crystal violet
DDW: Distilled deionized water
EGCG: Epigallocatechin gallate
EPS: Exopolysaccharides, extracellular polysaccharides
FTF: Fructosyltransferase
GTF: Glucosyltransferase
GTP: Green tea polyphenols
OD: Optical density
PBS: Phosphate buffered saline
qPCR: Quantitative polymerase chain reaction
QS: Quorum sensing
RT: Room temperature
*S. mutans*: *Streptococcus mutans*
HR-SEM: High resolution scanning electron microscopy
