# Supplementary material for: Effect of epigallocatechin gallate on dental biofilm of Streptococcus mutans: An in vitro study
Source: BMC Oral Health. 2021 Sep 15;21:447. doi: 10.1186/s12903-021-01798-4 (PMC8444437; doi:10.1186/s12903-021-01798-4)
Supplement: Supplementary file 1 — Additional file 1. Table 1: The primers used for real-time PCR. [file 12903_2021_1798_MOESM1_ESM.pdf]

Supplementary data

Effect of epigallocatechin gallate on dental biofilm of *Streptococcus mutans:* An *in vitro* study

Mor Schneider-Rayman, Doron Steinberg, Ronit Vogt Sionov, Michael Friedman, Miriam Shalish

Table 1. The primers used for real-time PCR.

Gene	Description	Primer Sequence (5'->3')	
		**Forward**	**Reverse**
gtfB	Glucosyltransferase enzymes, glucan production	AGCAATGCAGCCAATCTACAAAT	ACGAACTTTGCCGTTATTGTCA
gtfC		CTCAACCAACCGCCACTGTT	GGTTTAACGTCAAAATTAGCTGTATT
ftf	Fructosyltransferase enzyme, fructan production	AAATATGAAGGCGGCTACAACG	CTTCACCAGTCTTAGCATCCTGAA
brpA	Biofilm-regulatory protein	GGAGGAGCTGCATCAGGATTC	AACTCCAGCACATCCAGCAAG
luxS	Universal quorum sensing	ACTGTTCCCCTTTTGGCTGTC	AACTTGCTTTGATGACTGTGGC
recA	Homologous recombination of chromosomal DNA	AGG TGC AGT TAT GCG TCT GG	GCA ACA GCA TGA AGA GCG AC
nox	NADH oxidation reduction	GGGTTGTGGAATGGCACTTTGG	CAATGGCTGTCACTGGCGATTC
sodA	Superoxidae dismutase	GCAGTGCTAAGACTCCCGAATC	TTGCGGAAGTGTGAGATTGGC
vicR	Cytoplasmic response regulator –modulate gene expression	TGACACGATTACAGCCTTTGATG	CGTCTAGTTCTGGTAACATTAAGTCCAATA
spaP	Cell surface antigen	GACTTTGGTAATGGTTATGCATCAA	TTTGTATCAGCCGGATCAAGTG
groEL	Tolerance to environmental stresses	CCAGGAGCTTTGACTGCGAC	TTGCGGATGATGATGTAGATGGT
dnaK	Tolerance to environmental stresses	GCAGGTCAAGAGGGAGCTCA	CCGCCCTTGTCTGAGAATC
16S rRNA	Normalizing internal standard	CCTACGGGAGGCAGCAGTAG	CAACAGAGCTTTACGATCCGAAA
23S rRNA		GTGACGGGGAGCGAAGTT TA	TCACTGCGGCTGACTTATCG
